# Crystal Structural Characteristics and Electrical Properties of (Ba_0.7_Sr_0.3-*x*_Ca*_x_*)(Ti_0.9_Zr_0.1_)O_3_ Ceramics Prepared Using the Citrate Gelation Method

**DOI:** 10.3390/ma16247551

**Published:** 2023-12-07

**Authors:** Jae-Young Jeong, Si-Hyun Kim, Ju-Hye Kim, Jae-Hoon Park, Da-Som Jung, Eung-Soo Kim

**Affiliations:** Department of Advanced Materials Engineering, Kyonggi University, Suwon 16227, Republic of Korea; 20211101119@kyonggi.ac.kr (J.-Y.J.); sh.kim@kyonggi.ac.kr (S.-H.K.); jadoit7@kyonggi.ac.kr (J.-H.K.); hoonbasket@kyonggi.ac.kr (J.-H.P.); som9624@kyonggi.ac.kr (D.-S.J.)

**Keywords:** BaTiO_3_, citrate gelation, B-site bond valence, breakdown voltage

## Abstract

The electrical properties of (Ba_0.7_Sr_0.3-*x*_Ca*_x_*)(Ti_0.9_Zr_0.1_)O_3_ (0 ≤ *x* ≤ 0.2) (BSCTZ) ceramics prepared using citrate gelation (CG) method were investigated by substituting Ca^2+^ ions for the Sr^2+^ sites based on the structural characteristics of the ceramics. BSCTZ was sintered for 3 h at 1300 °C, lower than the temperature (1550 °C) at which the specimens prepared using the solid-state reaction (SSR) method were sintered, which lasted for 6 h. As the amount of substituted Ca^2+^ ions increased, the unit cell volume of the BSCTZ decreased because of the smaller ionic radius of the Ca^2+^ ions compared to the Sr^2+^ ions. The dielectric constant of BaTiO_3_-based ceramics is imparted by factors such as the tetragonality and B-site bond valence of the ceramics. Although the ceramic tetragonality increased with Ca^2+^ ion substitution, the *x* = 0.05 specimens exhibited the highest dielectric constant. The decrease in the dielectric constant of the sintered *x* > 0.05 specimens was attributed to the increase in the B-site bond valence of the ABO_3_ perovskite structure. Owing to the large number of grain boundaries, the breakdown voltage (6.6839 kV/mm) of the BSCTZ prepared using the CG method was significantly improved in relation to that (2.0043 kV/mm) of the specimen prepared using the SSR method.

## 1. Introduction

With the development of self-driving and electric vehicles, the demand for multilayer ceramic capacitors (MLCCs) in the automotive industry has rapidly increased [1]. Automotive MLCCs must exhibit high capacitance and are expected to be highly reliable in terms of withstanding harsh external environmental conditions, such as external shocks, high temperatures, and high levels of humidity. Hence, strategies for developing automotive MLCCs with both high capacitance and reliability must be explored [2].

BaTiO_3_ (BT) ceramics are piezoelectric materials frequently used to replace lead zirconium titanate (PZT) for a wide range of industries [3]. Many studies have investigated ways to improve the piezoelectric characteristics of BT, which has an inferior piezoelectric constant to that of PZT, such as controlling the grain size and employing other synthesis techniques [4]. In addition, due to the piezoelectric characteristics of BT, it can cause signal distortion and damage electronic devices when BT is used as dielectric layer of MLCC. It has been reported that since the piezoelectric response is dependent on the thickness of the BT layer, the piezoelectric response could be reduced by having the BT layer at the micron level [5].

Furthermore, BaTiO_3_ ceramics, which are ferroelectric dielectrics with ABO_3_ perovskite structures, have been used as base materials in capacitors because of their superior dielectric properties [6]. These ceramics exhibit various crystal structural phases depending on the displacement of the ions constituting the unit lattices of their crystal structures. Various dopants that can increase the dielectric constant (*ε_r_*) of BaTiO_3_ ceramics have been investigated [7]. The investigation of ideal dopant systems is necessary because dopants can increase both the tetragonality (c/a) and octahedral volume of a unit cell [8].

To apply BaTiO_3_-based ceramics in automotive MLCCs, the crystal structural characteristics of the ceramics must be improved and the uniformity of their microstructures need to be controlled [9]. These steps are crucial because a heterogeneous microstructure can reduce the reliability and capacitance of an electronic device. To enhance the microstructural uniformity of BaTiO_3_-based ceramics, various aspects of BaTiO_3_, such as synthesis procedures [10], sintering conditions [11], and nanostructural properties [12], have been studied. In particular, many researchers have investigated advanced synthetic methods, including the solid-state reaction (SSR) and sol–gel method, to prepare BaTiO_3_ powder with fine particles and high crystallinity [13].

The SSR method is commonly used in simple powder manufacturing processes because of its low raw material cost. However, it typically requires high sintering temperatures and long sintering times for the reaction to complete, and the resulting powders tend to contain large particles. Unlike the SSR method, the sol–gel method can effectively synthesise ceramic powders with high purity, small particle size, and good homogeneity at low temperatures [14].

The citrate gelation (CG) method is a liquid synthesis method that is a combination of the SSR and sol–gel methods. It is a simple method that produces uniform particles. In the CG method, a solid polymer network is formed via gelation to uniformly disperse Ti particles [15]. After calcination, the process is optimised using the SSR method, and cold isostatic pressing (CIP) is performed to synthesise low-loss dielectric ceramics. Through the CG method, ceramic powders with a smaller particle size and a more homogeneous microstructure than those prepared using the SSR method can be achieved because of the formation of nuclei in the liquid phase [16].

For the (Ba_0.7_Sr_0.3-*x*_Ca*_x_*)(Ti_0.9_Zr_0.1_)O_3_ (BSCTZ) (0 ≤ *x* ≤ 0.2) ceramics prepared using the SSR method, a high sintering temperature (1550 °C) and a low breakdown voltage (BDV*;* ~2 kV/mm) were reported [17]. Thus, the CG method was applied for the synthesis of BSCTZ ceramics in this study to achieve a low sintering temperature and increase the BDV. The dependence of the crystal structural and the microstructural characteristics of the BSCTZ ceramics prepared using the CG method on Ca^2+^ ion substitution was investigated. Additionally, the electrical properties, such as dielectric constant, dielectric loss, and BDV, of the BSCTZ ceramics, as well as the temperature coefficient of capacitance (*TCC*), were investigated.

## 2. Materials and Methods

The BSCTZ ceramics were prepared using high-purity barium nitrate (99.0%, Daejung, Busan, Republic of Korea), calcium nitrate (98.5%, Kanto, Tokyo, Japan), strontium nitrate (98.0%, Kanto, Tokyo, Japan), titanium isopropoxide (TTIP; 98.0%, Junsei, Tokyo, Japan), zirconium oxychloride (99.0%, Kanto, Tokyo, Japan), propylene glycol (PG; 99.5%, Daejung, Busan, Republic of Korea), and citric acid anhydrous (99.5%, Junsei, Tokyo, Japan) via the CG method.

TTIP and PG were weighed at a molar ratio of 1:1 and mixed at 50 °C for 24 h to disperse TTIP in PG. Barium nitrate, calcium nitrate, strontium nitrate, and zirconium oxychloride octahydrate were weighed with titanium-containing precursors at the stoichiometric ratios to obtain the required BSCTZ composition, after which they were mixed in distilled water to facilitate precursor hydrolysis. Thereafter, the TTIP and BSCTZ solutions were mixed; citric anhydrous acid (99.9%, Junsei, Tokyo, Japan) and distilled water (99.9%, Daejung, Busan, Republic of Korea) were added to the mixed solution and stirred at 80 °C for 24 h to enable the polyester reaction. The resulting solution was transformed into a gel by increasing the hot plate temperature to 350 °C. The organic compound obtained was sequentially heat-treated at 500 and 900 °C for 5 and 24 h, respectively, to remove residual carbon. The obtained powder was ball-milled for 24 h with stabilised ZrO_2_ balls in ethyl alcohol (99.9%, Daejung, Busan, Republic of Korea), followed by drying. The dried powders were pressed into pellets 15 mm in diameter via CIP under a pressure of 1500 kg/cm^2^. The prepared pellets were sintered at a temperature between 1300 and 1400 °C for 3 h in an air atmosphere.

For the phase identification of the BSCTZ ceramics, X-ray diffraction (XRD; Rigaku D/Max-3C, Tokyo, Japan) and Raman spectroscopy (HEDA, NOST system, Busan, Republic of Korea) equipped with a 532 nm excitation wavelength laser was conducted, while their crystal structural characteristics were determined through the Rietveld refinement (RIR) analysis using the Fullprof program (version. 7.95) [18]. Microstructural analysis of the sintered specimens was performed through scanning electron microscopy (SEM; JEOL JSM-6500F, Seoul, Republic of Korea). For the observed microstructure, the average grain size of the BSCTZ ceramics was obtained using the linear intercept method from the 5 SEM micrographs for each composition [19]. Silver electrodes were applied to the two surfaces of each sintered specimen, followed by firing at 650 °C for 10 min. The dielectric constant and dielectric loss of each specimen were measured using a precision impedance analyser (Agilent 4294A, Santa Clara, CA, USA). An electrical breakdown test was performed at room temperature by immersing the specimens in a silicone oil bath and applying a voltage, which increased at a rate of 0.02 kV/s, using a thin-film analyser (TF analyser, aixACCT systems 1000, Seoul, Republic of Korea). Additionally, the *TCC* was tested in a range from −35 to 85 °C at 1 kHz using a TCC measurement system (Nanoionics Korea TM-100, Seoul, Republic of Korea).

## 3. Results and Discussion

### 3.1. Physical Properties of BSCTZ Ceramics Prepared Using the CG Method

**Figure 1:**
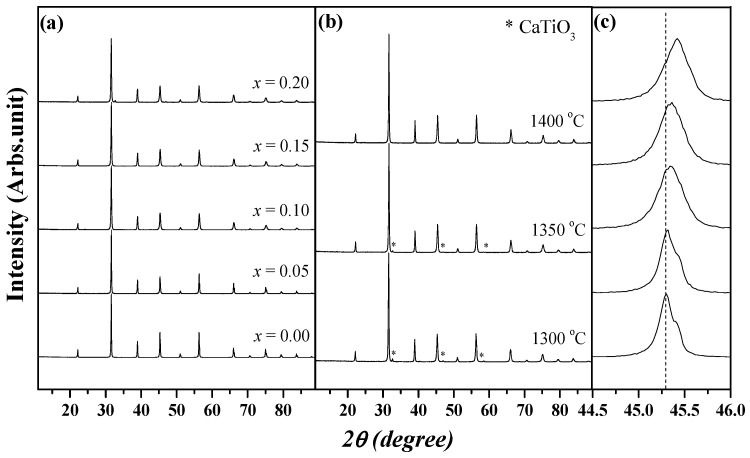
(**a**) X-ray diffraction (XRD) patterns of the (Ba_0.7_Sr_0.3-_*_x_*Ca*_x_*)(Ti_0.9_Zr_0.1_)O_3_ (BSCTZ) (0 ≤ *x* ≤ 0.2) ceramics sintered at 1300 °C for 3 h. (**b**) XRD patterns of the BSCTZ (*x* = 0.2) ceramic sintered at temperatures between 1300 and 1400 °C for 3 h. (**c**) Enlarged XRD patterns of BSCTZ ceramics sintered under optimal conditions.

The XRD patterns of the BSCTZ ceramic specimens sintered at 1300 °C for 3 h are shown in Figure 1a. A single BaTiO_3_ phase with a perovskite structure was observed for all compositions, except for the *x* = 0.2 specimen. In the specimen with *x* = 0.2 that was sintered at 1300–1350 °C for 3 h, a secondary phase of CaTiO_3_ was detected in the 2θ range of 30°–60°. The formation of the CaTiO_3_ secondary phase was controlled by increasing the sintering temperature to 1400 °C, as shown in Figure 1b. Figure 1c shows the enlarged XRD patterns of BSCTZ ceramics. With the increase in Ca^2+^ substitution, the diffraction peak of the (200) plane shifted to higher angle. The Ca^2+^ ion substitution in the BT lattice contributed to this.

**Figure 2:**
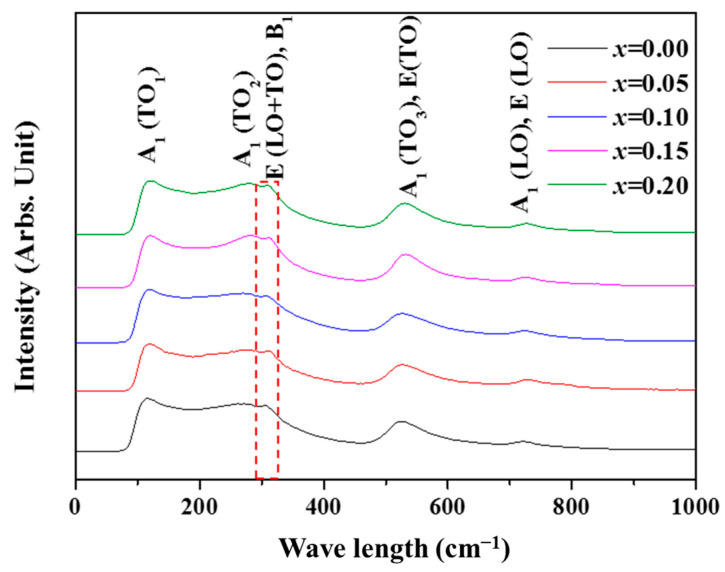
Raman spectra of the (Ba_0.7_Sr_0.3-_*_x_*Ca*_x_*)(Ti_0.9_Zr_0.1_)O_3_ (BSCTZ) (0 ≤ *x* ≤ 0.2) ceramics sintered under optimal conditions.

The Raman spectra of BSCTZ ceramics sintered at optimal sintering condition are shown in Figure 2. All of the BSCTZ ceramics show the band spectra around 123, 282, 305, 519, and 712 cm^−1^. The tetragonal structure of BaTiO_3_ shows three A_1_ + E (TO + LO) split from F_1U_ and B1 (TO + LO) split from F_2u_, which is partially due to the polarization from the Ti^4+^ and O^2-^ ions [20,21]. This means that BSCTZ ceramics have the tetragonal structure of BT.

**Figure 3:**
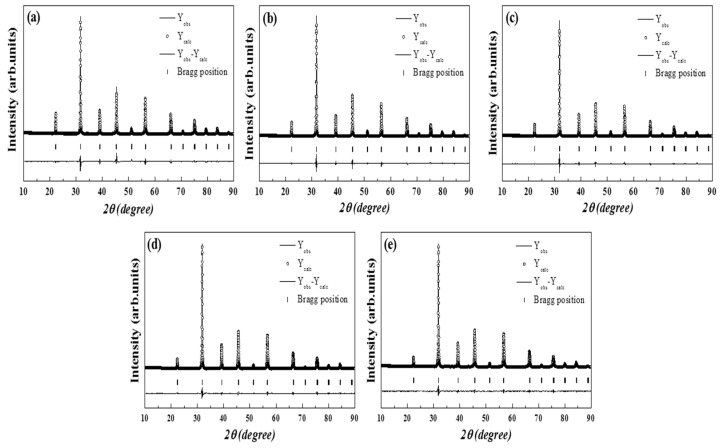
Observed and calculated Rietveld refinement patterns and the difference patterns of the BSCTZ ceramics. (**a**) *x* = 0.0. (**b**) *x* = 0.05. (**c**) *x* = 0.10. (**d**) *x* = 0.15, sintered at 1300 °C for 3 h. (**e**) *x* = 0.2, sintered at 1400 °C for 3 h.

To investigate the detailed structural characteristics of the 0 ≤ *x* ≤ 0.15 specimens sintered at 1300 °C for 3 h and those of the *x* = 0.2 specimens sintered at 1400 °C for 3 h, Rietveld refinement was performed on the basis of the crystal symmetry of tetragonal (P4 mm) BaTiO_3_ from the Raman spectroscopy result, as shown in Figure 3. The dotted and solid lines in the figure denote the observed and calculated intensities of the values of two intensities.

The specific crystal structure refinement results and R-factors are listed in Table 1 for each sintered specimen. The goodness of fit (Gof) and Bragg R-factor, which is the reliability indicator of RIR, were within the ranges of 1.9–2.5 and 1.52–3.82, respectively. These values indicate that the refined results are reliable. The unit cell volumes of the sintered specimens decreased with Ca^2+^ ion substitution, owing to the smaller ionic radius of the Ca^2+^ ion (1.34 Å, coordination number (CN) = 12) than that of the Sr^2+^ ion (1.44 Å, CN = 12) [22].

**Figure 4:**
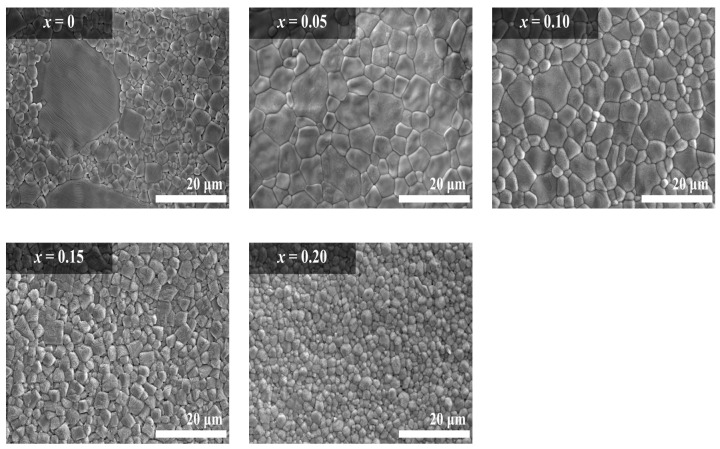
SEM micrographs of the BSCTZ ceramics sintered under optimal sintering conditions. (scale bar = 20 μm).

The SEM images of the BSCTZ ceramics are shown in Figure 4. The BSCTZ ceramics exhibited a dense microstructure with a high relative density (>93% of the theoretical density), as indicated by the values listed in Table 1. For the BSTZ ceramic (*x* = 0.00), abnormal grain growth with {111} twins (dark region) was detected, which is a similar result to the previous studies [23,24]. For all specimens, the average grain size was significantly smaller than that of the BSCTZ specimen prepared using the SSR method [17]. The average grain size of the BSTZ ceramic (*x* = 0.00) was 1.243 μm, while the BSCTZ ceramic (*x* = 0.05) showed a large average grain size (1.822 μm). For the BSCTZ specimen (0.10 ≤ *x* ≤ 0.20) with high Ca^2+^ substitution, the average grain size decreased with the Ca^2+^ content from 1.231 μm to 0.792 μm. This behaviour can be attributed to the smaller ionic radius of the Ca^2+^ ion (1.34 Å, CN = 12) compared to that of the Sr^2+^ ion (1.44 Å, CN = 12), as reported by Borkar et al. [25].

### 3.2. Electrical Properties of BSCTZ Ceramics Prepared by the CG Method

**Figure 5:**
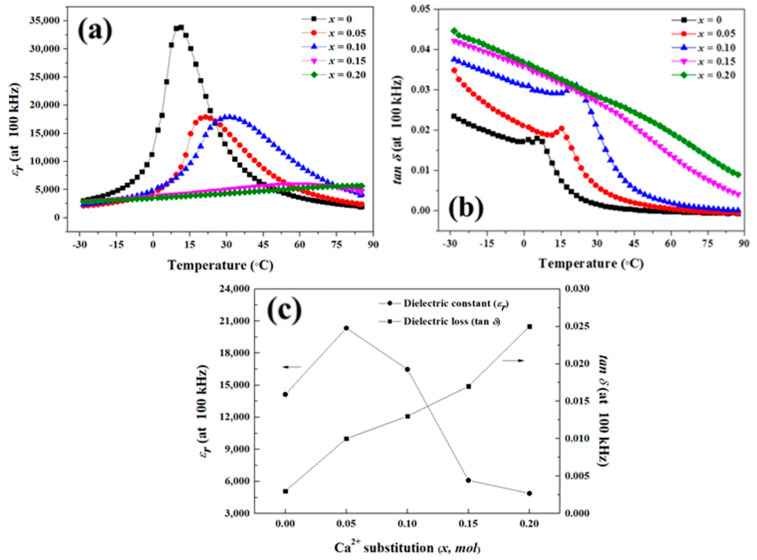
Temperature dependence of the (**a**) dielectric constant (ε_r_) and (**b**) dielectric loss (tan δ). (**c**) Dielectric constant (ε_r_) and dielectric loss (tan δ) of the BSCTZ ceramics sintered under optimal sintering conditions and measured at room temperature (@ 25 °C).

Figure 5 shows the dependence of the dielectric constant and dielectric loss of the specimens on the temperatures; these parameters were measured at 100 kHz. Figure 5a shows the temperature dependence of the dielectric constant of the specimens. Since Ca^2+^ ions act as depressors and Sr^2+^ ions act as shifters, the Curie temperature of the BSCTZ ceramics was increased with the increase in Ca^2+^ ion substitution [26,27]. Also, the rate of change in capacitance according to the temperature decreased. Figure 5b demonstrates the temperature dependence of the dielectric loss of the specimens. The dielectric loss of the sintered specimens decreased as the temperature increased. At low temperatures, the domain wall movement increased the friction and used increased amount of energy, resulting in a high dielectric loss. At high temperatures, the dipoles with high energy rearranged easily owing to thermal disturbances, leading to a low dielectric loss [28]. Thus, the dielectric loss decreased as the temperature increased. With the increase in Ca^2+^ ion substitution, the dielectric loss of the sintered specimens increased due to the increase in grain boundaries, as confirmed in Figure 4 [29]. Figure 5c shows the dielectric constant and the dielectric loss measured at room temperature for the BSCTZ ceramics sintered under the optimal sintering conditions. The dielectric constant of the sintered specimens increased for the *x* = 0.05 specimen, but then decreased with further Ca^2+^ ion substitution. In contrast, the dielectric loss of the sintered specimens increased with Ca^2+^ ion substitution.

**Figure 6:**
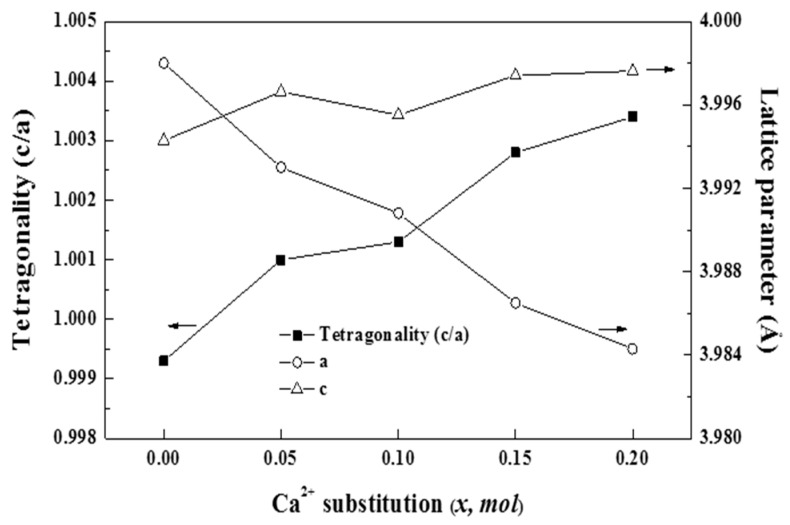
Tetragonality (c/a) and lattice parameter (Å) of the BSCTZ ceramics sintered under optimal sintering conditions.

The dielectric constants of BaTiO_3_-based ceramics depend on factors such as tetragonality and B-site bond valence. With an increasing amount of substituted Ca^2+^ ions, the a-axis of the sintered specimens changed significantly in relation with the c-axis, resulting in an increase in the tetragonality of the specimens, as shown in Figure 6. The tetragonality of BaTiO_3_-based ceramics at room temperature is related to their Curie point [30]. The increase in tetragonality with the increase in Ca^2+^ ion substitution was consistent with the shift in the Curie peak to a high temperature for the BSCTZ ceramics, as shown in Figure 5b and Figure 6. Furthermore, tetragonality is influenced by the lattice distortion resulting from ion substitution. As the tetragonality increases, the degree of lattice asymmetry also increases, leading to an increase in the dielectric constant [31]. Hence, the dielectric constant can be expected to increase with Ca^2+^ ion substitution. However, the *x* = 0.05 specimen showed the highest dielectric constant despite its tetragonality being lower than that of any other specimen. Thus, the influence of B-site bond valences on the dielectric constants of sintered specimens should be considered.

The dielectric properties of the BSCTZ ceramics were imparted by the displacement of the Ti^4+^ and/or Zr^4+^ ions from the centre of the [TiO_6_] octahedron, resulting in a local dipole moment. This dipole moment changed with the Ca^2+^ substitution based on its crystal symmetry and unit cell volume. The B-site bond valences of the BSCTZ ceramics with ABO_3_ perovskite structures were calculated using Equations (1) and (2) on the basis of the Rietveld refinement analysis results [32]. The results of the calculations are listed in Table 2.

(1) $$V_{B}=\sum_{j=1}^{N}\upsilon_{B-O}$$

(2) $$\upsilon_{B-O}=exp[(R_{B-O}-d_{B-O})/b]$$

where *R_B_*_−_*_O_* is the bond valence parameter, *d_B_*_−_*_O_* is the bond length between the B-site cations and oxygen ions, and *b* is a universal constant generally considered to be equal to 0.37 Å. The bond valence parameters closely match those reported by Brese and O’Keeffe [33]. The bond lengths were calculated on the basis of the Rietveld refinement analysis results.

**Figure 7:**
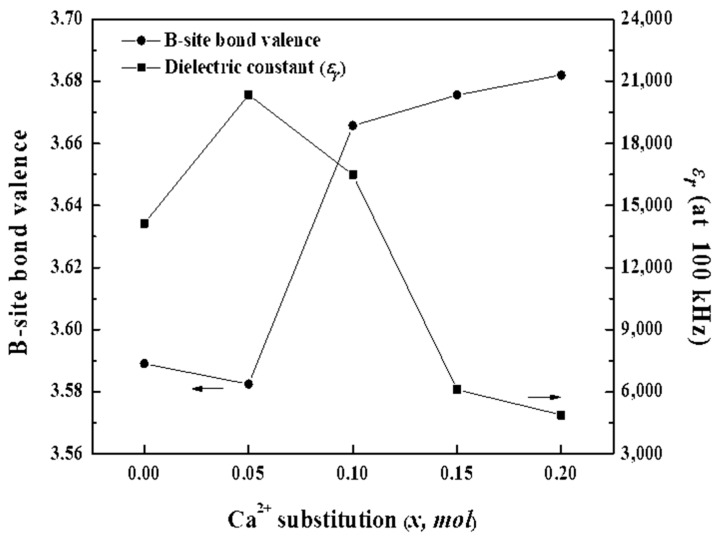
Dependence of the dielectric constant (ε_r_) on the B-site bond valence of BSCTZ ceramics sintered under optimal sintering conditions.

Figure 7 displays the dependence of the dielectric constants of the BSCTZ ceramics on the B-site bond valences. A low B-site bond valence implies weak bonding between the Ti^4+^ and constituent oxygen ions, resulting in enhanced vibration of the B-site ions in the oxygen octahedron. Thus, the dielectric constant increases due to the displacement of the Ti^4+^ ions from the [BO_6_] octahedron’s centre [34]. Accordingly, the *x* = 0.05 specimen showed the highest dielectric constant, while its B-site bond valence was the lowest.

As shown in Figure 5c, with the increase in the amount of substituted Ca^2+^ ions, the dielectric loss of the BSCTZ ceramics measured at 100 kHz showed an increase from 0.003 to 0.025. This dielectric loss is higher than that measured at 100 kHz in a previous study (0.001–0.007) on BSCTZ ceramics prepared using the SSR method. This result is consistent with the decrease in grain size and the increase in the number of grain boundaries [29].

**Figure 8:**
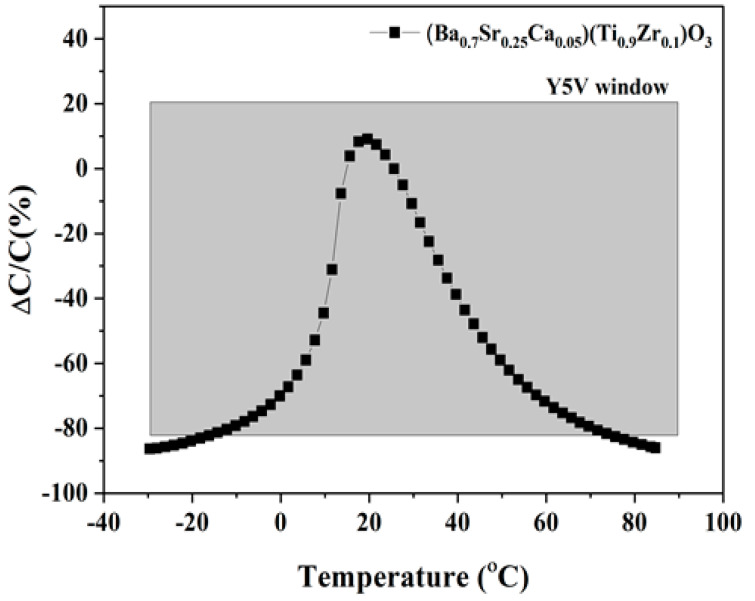
Temperature coefficient of capacitance of the *x*= 0.05 specimen at 1 kHz.

To evaluate the temperature stability of the capacitances of the BSCTZ ceramics, their *TCC* values were measured at temperatures ranging from −35 to 85 °C (Figure 8). The *x* = 0.05 specimen met the Y5V standard of the Electronic Industries Association (EIA), with a shift in the capacitance between −82% and +22%.

**Figure 9:**
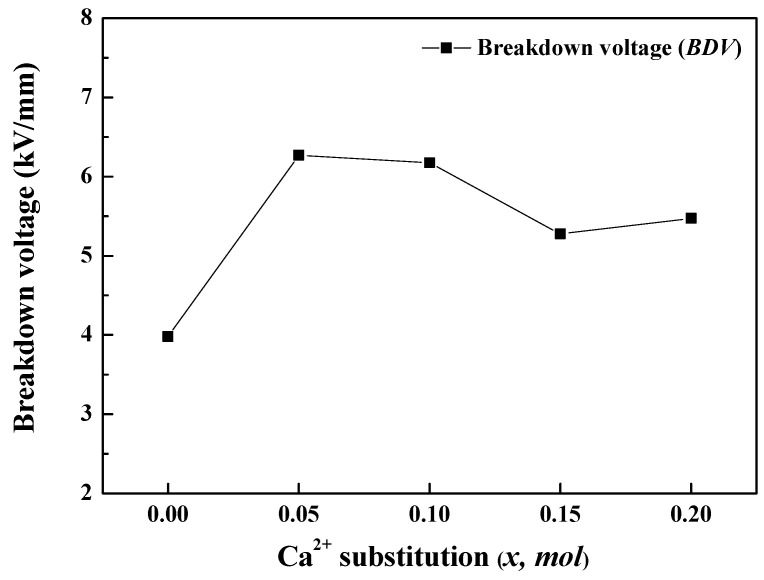
Breakdown voltage of the BSCTZ ceramics sintered under optimal sintering conditions.

The BDV values of the BSCTZ ceramics sintered under the optimal sintering condition and prepared using the CG method are shown in Figure 9. For all sintered specimens, the BDV values of the BSCTZ ceramics prepared using the CG method were higher than those prepared using the SSR method. In particular, the BDV value (=6.6839 kV/mm) of the *x* = 0.05 specimen prepared using the CG method was three times higher than that (=2.0043 kV/mm) of the *x* = 0.05 specimen prepared by the SSR method. This high value can be attributed to the characteristics of the CG method, which induces nucleation during powder production in the liquid phase, resulting in smaller grain sizes and a higher number of grain boundaries than those prepared through the SSR method [17].

## 4. Conclusions

The dependence of the crystal structural characteristics and dielectric properties of the BSCTZ ceramics prepared using the CG method with Ca^2+^ ion substitution was investigated. As the amount of substituted Ca^2+^ ions increased, the unit cell volumes of the sintered specimens decreased because of the smaller ionic radius of the Ca^2+^ ion compared to that of the Sr^2+^ ion. The tetragonality and B-site bond valence of each specimen were calculated using the Rietveld refinement analysis; the tetragonality increased as the amount of the substituted Ca^2+^ ions increased. Moreover, the grain size of each specimen decreased and its dielectric loss increased with Ca^2+^ ion substitution. The BDV values of the specimens prepared using the CG method improved significantly in relation to those using the SSR method because of the increase in the number of grain boundaries due to the formation of nuclei in the liquid phase. The *x* = 0.05 specimen exhibited the highest dielectric constant and met the Y5V standard of the EIA.

## Figures and Tables

**Table 1:** Rietveld refinement results of the BSCTZ ceramics sintered under optimal sintering conditions.

BSCTZ (*x*)	Sintering Condition (°C/h)	Crystal Structure (Space Group)	Unit Cell Volume (Å^3^)	Tetragonality (c/a)	GoF	R_Bragg_	Relative Density (%)
0.00	1300 °C/3 h	Tetragonal (P4 mm)	63.8547	0.9993	2.5	1.95	98.08
0.05			63.7305	1.0010	2.4	2.86	97.22
0.10			63.6425	1.0013	2.3	1.52	93.66
0.15			63.5354	1.0028	1.9	3.82	95.67
0.20	1400 °C/3 h		63.4677	1.0034	2.0	1.72	95.08

**Table 2:** Calculated B-site bond valences of the BSCTZ ceramics sintered under optimal sintering conditions.

BSCTZ (*x*)	Sintering Condition (°C/h)	*d_Ti_*_−_*_O_* (1) (Å)	*d_Ti_*_−_*_O_* (2) (Å)	*d_Ti_*_−_*_O_* (3) (Å)	B-Site Bond Valence
0.00	1300 °C/3 h	1.9975	2.009	2.0061	3.5891
0.05		1.9986	2.010	2.0066	3.5824
0.10		1.9979	1.997	1.9972	3.6657
0.15		1.9990	1.995	1.9960	3.6756
0.20	1400 °C/3 h	1.9991	1.994	1.9952	3.6820

## Data Availability

Data are contained within the article.
